# Material for Men

**Published:** 1857-11

**Authors:** 


### MATERIAL FOR MEN.

A LONG time ago, a little boy twelve years old, on his road to Vermont, stopped at a country tavern, and paid for his lodging and breakfast by sawing wood, instead of asking for it as a gift. Fifty years later, the same boy passed that same little inn as GEORGE PEABODY, the banker, whose name is the synonyme of magnificent charities—the honored of two hemispheres.

Far back in the teens of the present century, a young man asked for employment in the Springfield armory; but he was poor, and modest, and had no friends, so he went away without it; but, feeling the man within him, he sought work until he found it. An age later, he visited that armory the second time, not as a common day-laborer, but as the ablest speaker of the House of Representatives for many years, and not unlikely our future chief magistrate—N. P. BANKS, of New Hampshire. Go to, ye dandified, kid-gloved, cologned, billiard-playing, club-tending, tight-waisted, spindle-limbed, soft-headed, time killing, lady-tending numskulls, begin life over again, if you wish to leave your mark on the world's history, by earning your first dinner in honorable labor; and resolve, that what you eat and drink, and wear, from this day forward, shall be from your own earnings, and you will yet die men.

